# Biomass Smoke Exposure Is Associated With Gastric Cancer and Probably Mediated Via Oxidative Stress and DNA Damage: A Case-Control Study

**DOI:** 10.1200/GO.20.00002

**Published:** 2020-03-31

**Authors:** Violet Kayamba, Kanekwa Zyambo, Chola Mulenga, Simutanyi Mwakamui, Mizinga Jacqueline Tembo, Aaron Shibemba, Douglas Corbett Heimburger, Masharip Atadzhanov, Paul Kelly

**Affiliations:** ^1^ Department of Internal Medicine, Tropical Gastroenterology and Nutrition Group, Lusaka, Zambia; ^2^ Department of Internal Medicine, University of Zambia School of Medicine, Lusaka, Zambia; ^3^ University of Zambia School Health Sciences, Lusaka, Zambia; ^4^ Department of Pathology, University Teaching Hospital, Lusaka, Zambia; ^5^ Vanderbilt Institute for Global Health, Vanderbilt University Medical Center, Nashville, TN; ^6^ Blizard Institute, Barts and The London School of Medicine and Dentistry, Queen Mary University of London, London, United Kingdom

## Abstract

**PURPOSE:**

We investigated the association between gastric cancer and environmental and dietary exposures. In addition, we explored probable mechanistic pathways for the influence of biomass smoke on gastric carcinogenesis.

**PATIENTS AND METHODS:**

The study was conducted in Lusaka, Zambia. Questionnaires were used to collect data on risk factors, whereas enzyme-linked immunosorbent assays and high-performance liquid chromatography were used to measure biologic exposures. Study data were analyzed using contingency tables and logistic regression.

**RESULTS:**

We enrolled 72 patients with gastric adenocarcinoma and 244 controls. Gastric cancer was positively associated with rural residence (odds ratio [OR], 2.9; 95% CI, 1.5 to 5.3), poverty (OR, 4.2; 95% CI, 1.9 to 9.1), and daily consumption of processed meat (OR, 6.4; 95% CI, 1.3 to 32) and negatively associated with consumption of green vegetables (OR, 0.2; 95% CI, 0.1 to 0.5). Gastric cancer was also associated with biomass smoke exposure (OR, 3.5; 95% CI, 1.9 to 6.2; *P* < .0001), an association that was stronger for intestinal-type cancers (OR, 3.6; 95% CI, 1.5 to 9.1; *P* = .003). Exposure to biomass smoke in controls was associated with higher urinary levels of 8-isoprostane (*P* < .0001), 8-hydroxydeoxyguanosine (*P* = .029), and 1-hydroxypyrene (*P* = .041). Gastric cancer was not associated with biochemical measures of current exposure to aflatoxins or ochratoxins.

**CONCLUSION:**

In Zambia, exposure to biomass smoke, daily consumption of processed meat, and poverty are risk factors for gastric cancer, whereas daily consumption of green vegetables is protective against gastric cancer. Exposure to biomass smoke was associated with evidence of oxidative stress and DNA damage, suggesting mechanistic plausibility for the observed association, and the association was restricted to intestinal-type gastric cancer.

## INTRODUCTION

Gastric cancer is one of the leading causes of cancer-related deaths worldwide,^1^ but in Africa, data on its occurrence are scanty.^2^ Scarce resources limit the ability to diagnose, treat, and stage gastric cancer, compromising the ability to clearly understand the epidemiology of the disease in Africa.^3^ Furthermore, there are limited population-based registries and poor record keeping in many African centers.^4^ Our preliminary work on gastric cancer in Zambia provided the basis to further explore risk factors associated with gastric carcinogenesis. We observed that the diagnosis of early-onset gastric cancer was unexpectedly high and that patient outcomes were poor.^5-7^ These preliminary data also showed no association between gastric cancer and the *Helicobacter pylori* virulence factor CagA.^6,8^ Therefore, we endeavored to evaluate environmental and dietary influences on gastric cancer in Zambia.

The role of nutrition in the development of gastric cancer has been widely investigated with inconsistent results,^9^ and much of the data have been obtained from observational studies.^10^ Mycotoxins are one of the most common dietary contaminants in Africa.^12-14^ They are small-molecular-weight compounds produced by filamentous fungi or molds under suitable temperatures and humidity. Aflatoxins are such toxins, produced by fungi of the *Aspergillus* species and known to contaminate peanuts and maize, whereas ochratoxins mainly contaminate cereals, coffee, and grape berries. These mycotoxins have the potential to be carcinogenic. Biomass smoke is produced by the combustion of any organic matter, such as firewood, charcoal, grass, or dung. These are common sources of fuel in Africa, with > 70% of the population depending on biomass fuels.^15^ However, the health consequences of long-term, or even lifetime, exposure to biomass smoke have been poorly investigated. Several researchers have linked squamous cell carcinoma of the esophagus to biomass smoke exposure,^16-18^ but the evidence for gastric cancer is scanty. For example, a study involving fire fighters in Sweden showed that they had an increased risk of developing gastric cancer,^19^ but this was indirect evidence.

**CONTEXT**

**Key Objective**
There is a high proportion of early-onset gastric cancer among Zambian adults. This cannot be explained by the high incidence of *Helicobacter pylori* infection alone. Therefore, we set out to investigate other factors that could be associated with gastric cancer.
**Knowledge Generated**
We found that exposure to biomass smoke, poverty, and rural residence were risk factors for gastric cancer. Exposure to biomass smoke on its own was associated with oxidative stress. We also found that daily consumption of green vegetables was negatively associated with gastric cancer. We found no association with either aflatoxin or ochratoxin exposure.
**Relevance**
Our study indicates that improvement of socioeconomic status, use of cleaner fuels, and healthier diets could affect the occurrence of gastric cancer in Zambia.

We explored a possible link of gastric carcinogenesis to dietary factors including exposure to mycotoxins and biomass smoke. In addition, we explored measurable biomarkers including urinary 1-hydroxypyrene (1-OHP), a metabolite of one of the major constituent groups of biomass smoke, polycyclic aromatic hydrocarbons. Evidence of oxidative stress measured by urinary 8-isoprostanes was also evaluated. Oxidative stress to DNA double bonds was estimated using urinary 8-hydroxydeoxyguanosine (8-OHdG) and serum γ-H2AX. H2AX is one of the histone octamers that DNA is wrapped around and could be used to estimate double-stranded breaks. Although some of these biomarkers reflect only short-term exposure, we looked for early evidence that they could illuminate the connection between exposure and cancer development. The University of Zambia Biomedical Research Ethics Committee (reference No. 000-03-16) approved this study.

## PATIENTS AND METHODS

### Patient Recruitment

This was a case-control study conducted at the University Teaching Hospital (UTH) in Lusaka, Zambia, between July 2016 and April 2018. UTH is the largest referral hospital in Zambia, serving patients from all ten provinces of the country. Patients referred for a diagnostic esophagogastroduodenoscopy (EGD) were targeted for enrollment. Patient cases were patients diagnosed with gastric cancer, whereas controls had no endoscopic or histologic evidence of malignancy. All patients presenting to the UTH endoscopy unit for EGD were considered for study enrollment. Patients who gave written informed consent were included in the study. From these, we excluded individuals with prior history of gastric or esophageal cancer diagnosis or therapy. For assessment of lifestyle risk factors, we used interviewer-administered questionnaires, which included questions on diet, biomass smoke exposure, and socioeconomic factors. Biologic samples were collected to search for biochemical corroboration of reported exposures.

### EGD

After obtaining consent, an EGD was performed in fasted patients. During the EGD, biopsies were taken from gastric lesions suspected of being malignant. In all cases, at least six biopsies were taken from various sites of the lesions. For those without suspicious lesions, six biopsies were taken, two each from the antrum, incisura, and body. All biopsies were immediately placed in formalin and sent for histopathology. An experienced technician using standard methods processed biopsies sent for histopathology. The study histopathologist (A.S.) evaluated all the slides to determine the histologic diagnosis. Gastric adenocarcinomas were divided using the Lauren classification into intestinal type, diffuse type, and mixed type (composed of both the intestinal and diffuse types). Peripheral blood (serum later extracted) and urine samples were collected and stored at −80°C until analysis using the techniques outlined in the following sections.

### Urinary 8-OHdG

Urine levels of 8-OHdG were measured using enzyme-linked immunosorbent assay (ELISA) kits (MyBioSource, San Diego, CA) following the manufacturer’s instructions. Optical density was read at 450 nm. Results were corrected for creatinine excretion and reported as nanograms of 8-OHdG per milligram of creatinine.

### Urinary 8-Isoprostanes

Following the manufacturer’s instructions, urinary levels of 8-isoprostane were measured using ELISA (Detroit R&D, Detroit, MI). Optical density was read at 450 nm. These readings were also corrected for creatinine, and the final results were reported as nanograms of 8-isoprostanes per milligram of creatinine.

### Serum Human H2AFX

Sera were analyzed for human H2AFX (histone H2AX) by ELISA (MyBioSource) at 1:10 dilution, as instructed by the manufacturer. Optical density was again read at 450 nm, and the results are reported in picogram per milliliter.

### Urinary 1-OHP

High-performance liquid chromatography (HPLC) was used to measure urinary concentration of 1-OHP after enzymatic hydrolysis of the conjugates. The phenolic compound stock standard (Clincal; reference No. 9925; Recipe Chemicals, Munich, Germany) was used as the calibrator to make up standards from concentrations of 0.55 to 3.00 µg/L in deionized water. To all samples, calibrators, and controls (600 µL volume), β-glucuronidase enzyme mix (300 µL) was added. The enzyme mix was prepared by adding β-glucuronidase (50 µL) to 0.1 M of sodium acetate buffer pH 5 (5 mL). All samples, controls, and calibrators were incubated at 37°C for 4 hours followed by analysis on the HPLC system. Two certified reference controls (ClinChek; reference Nos. 8923 and 8924; Recipe Chemicals), levels 1 and 2, were used and run after the calibration and after every 10 samples. The percent recovery of the 2 certified reference controls, ClinCheck levels 1 and 2, are 97% and 95%, respectively. The limit of quantitation was 0.052 µg/L. A Waters (Milford, MA) system HPLC was used, with a 1525 binary pump, 717 autosampler, and 2475 fluorescence detector. The mobile phase consists of a methanol-to-water ratio of 3:1. The volume injected was 100 µL, and separation was performed on a Phenomenex SphereClone 3 µm ODS (2) 80 Å 100 × 4.6 mm column (Phenomenex, Torrance, CA). The excitation wavelength was set at 242 nm and the emission wavelength at 388 nm. The flow rate was 1 mL/min for 5 minutes. The levels of 1-OHP were reported in microgram per gram of creatinine.

### Testing for HIV

Serum was tested for HIV antibodies using Uni-Gold rapid diagnostic kits (Trinity Biotech, Wicklow, Ireland).

### Statistical Analysis and Data Availability

Medians and interquartile ranges were used to summarize continuous variables. Two-way analyses were used to look for associations between gastric cancer and the exposures of interest using either the Fisher’s exact or χ^2^ test. In addition, stepwise unconditional logistic regression was used to assess the relative contributions of different exposure variables. Odds ratios (ORs) were computed with 95% CIs. Nonparametric trend tests were used to assess ORs for ordered outcomes. The Kruskal-Wallis test was used to determine associations between various outcome continuous variables. *P* < .05 was considered statistically significant. Data were analyzed using STATA version 15 (Stata Corp, College Station, TX).

## RESULTS

**FIG 1:**
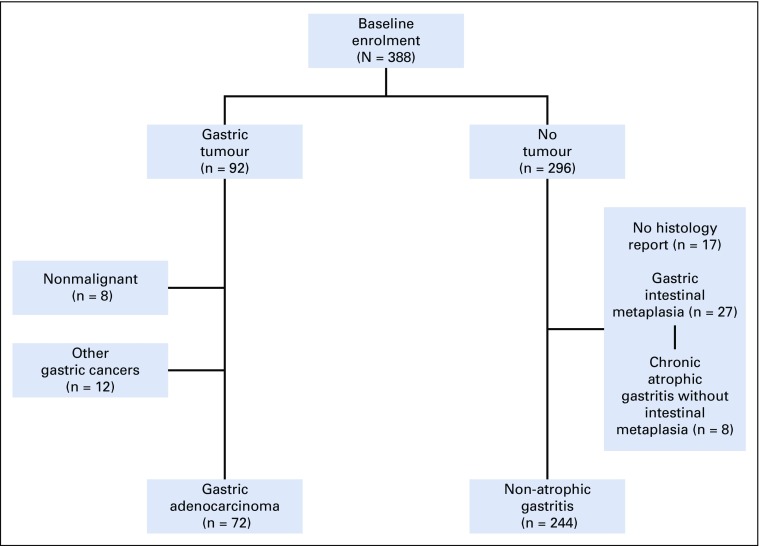
A flowchart showing the enrollment of patients into the study. Patients without histology reports, unconfirmed cancer, or cancer other than gastric adenocarcinoma were excluded from the analysis.

We recruited and studied 388 participants, 92 of whom (24%) had gastric cancer seen during endoscopy and 296 of whom were initially recruited as controls. For analysis of risk factors, only those with confirmed adenocarcinoma were included as patient cases (n = 72). The remaining 20 patients with gastric cancer seen during endoscopy had either no confirmatory histology report (n = 8) or other types of gastric cancer, including squamous cell or unclassified carcinomas (n = 8), gastric stromal cancer (n = 2), non-Hodgkin lymphoma (n = 1), or a hematolymphoid tumor (n = 1). Patients without any detectable gastric premalignant lesions seen on histology were used as controls (n = 244). We excluded patients with either gastric atrophy or intestinal metaplasia from the final risk factor analysis (Fig 1).

### Characteristics of Enrolled Patients and Their Association With Gastric Cancer

**FIG 2:**
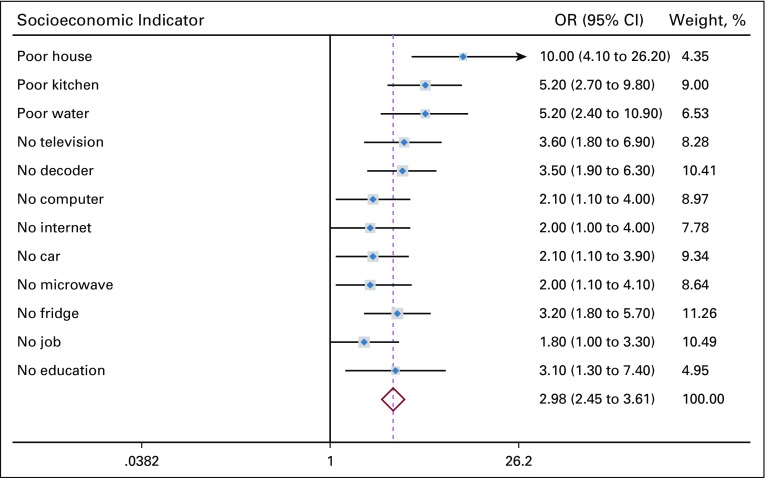
Socioeconomic indicators of gastric adenocarcinoma patients compared with controls. OR, odds ratio.

Patients with gastric cancer were significantly older than those without cancer (Table 1). Therefore, age was adjusted for in subsequent analyses. Gastric cancer was significantly associated with poor housing, poor water, and lack of a kitchen (all *P* < .05; Fig 2). Patients with gastric cancer had higher odds of having a low socioeconomic status assessed using the previously mentioned parameters (OR, 4.2; 95% CI, 1.9 to 9.1; *P* = .0002). Applying an unconditional logistic regression analysis adjusted for age, sex, and residence, patients with gastric cancer had significantly higher odds of not having good housing (OR, 5.3; 95% CI, 2.1 to 13.5; *P* < .0001) or a kitchen (OR, 3.4; 95% CI, 1.5 to 7.4; *P* = .003).

**TABLE 1:**
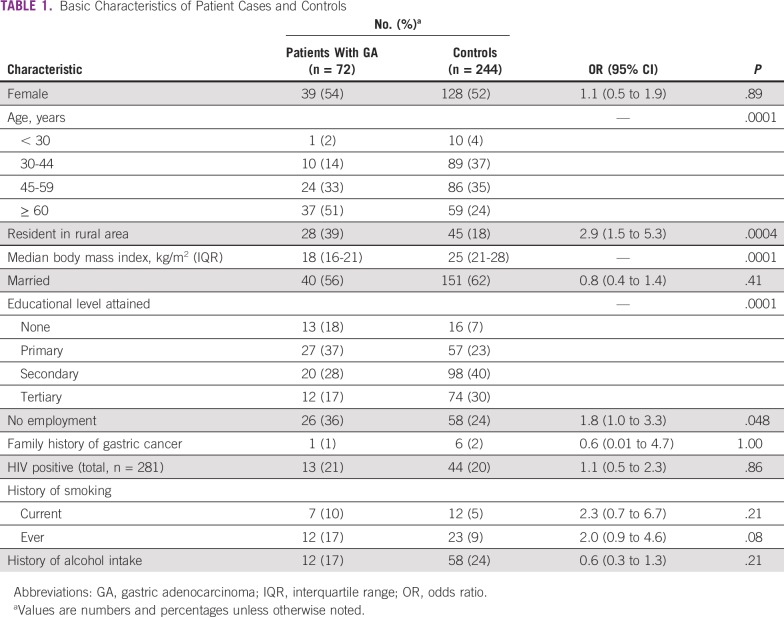
Basic Characteristics of Patient Cases and Controls

### Association Between Gastric Cancer and Biomass Smoke Exposure

Overall, 110 (35%) of 316 participants reported that they were completely reliant on biomass fuel for cooking, whereas another 107 (34%) of 316 used it occasionally because they had access to electric stoves. Thirty-nine percent of participants (99 of 316 participants) did not use biomass fuels in their homes at all. There was an association between gastric cancer and reliance on biomass fuel (OR, 3.5; 95% CI, 1.9 to 6.2; *P* < .0001). Two of the 3 unconditional logistic regression models factoring in parameters used to assess socioeconomic status and basic characteristics showed that biomass smoke exposure was an independent risk factor gastric cancer (Table 2). Of the patients with results for Lauren tumor classification, 28 (62%) of 45 had intestinal, 15 (34%) of 45 had diffuse, and 2 (4%) had mixed type of gastric cancer. Intestinal-type gastric cancer was associated with biomass smoke (OR, 3.6; 95% CI, 1.5 to 9.1; *P* = .003), whereas the diffuse type was not (OR, 0.9; 95% CI, 0.2 to 3.1; *P* = 1.00).

**TABLE 2:**
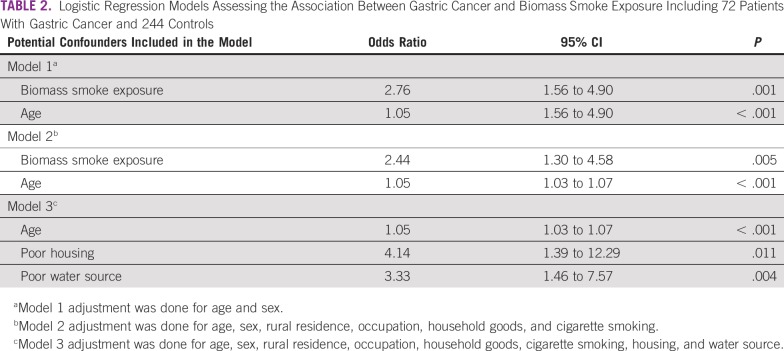
Logistic Regression Models Assessing the Association Between Gastric Cancer and Biomass Smoke Exposure Including 72 Patients With Gastric Cancer and 244 Controls

### Associations Between Biomass Smoke Exposure and Measurable Biomarkers in Patients Without Gastric Cancer

We compared levels of 1-OHP, 8-OHdG, γ-H2AX, and 8-isoprostanes between patients frequently, occasionally, and rarely exposed to biomass smoke. Patients with history of cigarette smoking (n = 19) were excluded from this analysis. The median 1-OHP level among the patient cases was 0.2 μg/g creatinine (interquartile range [IQR], 0.1-0.3 μg/g creatinine), and in the control group, it was 0.2 μg/g creatinine (IQR, 0.1-0.4 μg/g creatinine; *P* = .18). Urinary concentrations of 8-OHdG were higher among patient cases (median, 7.1 ng/mg creatinine; IQR, 3.2-20.8 ng/mg creatinine) than controls (median, 4.0 ng/mg creatinine; IQR, 2.0-10 ng/mg creatinine; *P* = .012).

**FIG 3:**
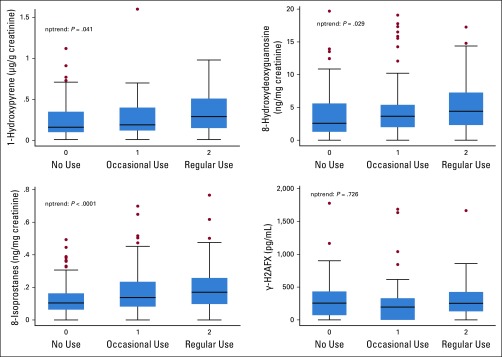
Association between biomass smoke exposure and urinary 8-hydroxydeoxyguanosine, 8-isoprostanes, 1-hydroxypyrene, and serum human H2AFX in patients without gastric cancer. nptrend, nonparametric test for trend.

We then analyzed the influence of biomass smoke exposure on these biomarkers among controls only. Using the nonparametric test for trend, measured levels of 1-OHP, 8-OHdG, and 8-isoprostanes were significantly higher with increased use of biomass fuels (Fig 3).

### Dietary Exposures and Gastric Cancer

Table 3 compares daily and regular (at least once a week) consumption of various food types and groups in patients with gastric cancer and controls. There was a significant negative association between gastric cancer and regular consumption of green vegetables, eggplants, or fruit (Table 3). Despite the small numbers, daily consumption of processed sausages was associated with gastric cancer. Using unconditional logistic regression adjusted for age, sex, and residence, gastric cancer was negatively associated with daily consumption of green vegetables (OR, 0.2; 95% CI, 0.1 to 0.5; *P* = .0001) and positively associated with daily consumption of processed meat (OR, 6.4; 95% CI, 1.3 to 31.8; *P* = .022).

**TABLE 3:**
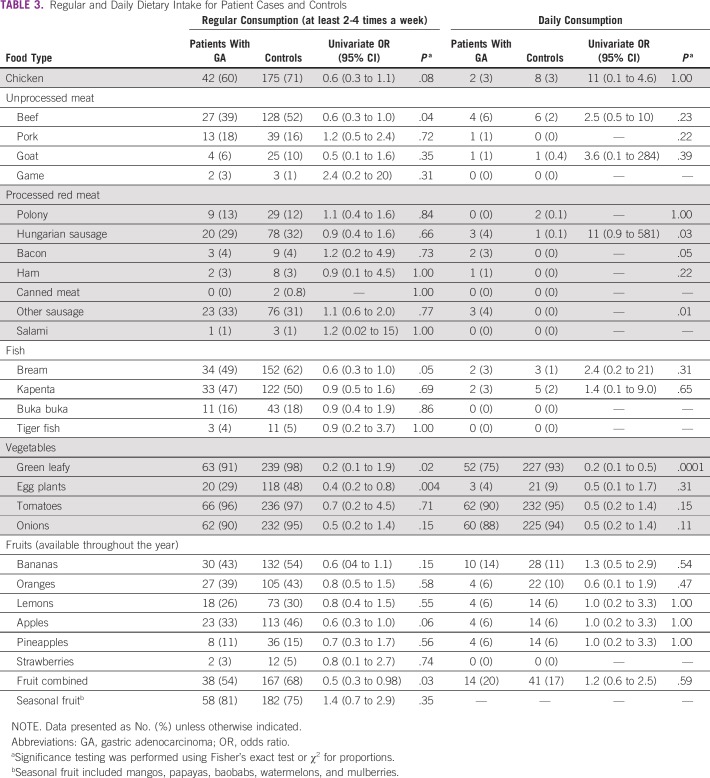
Regular and Daily Dietary Intake for Patient Cases and Controls

### Gastric Cancer and Dietary Exposure to Mycotoxins

**FIG 4:**
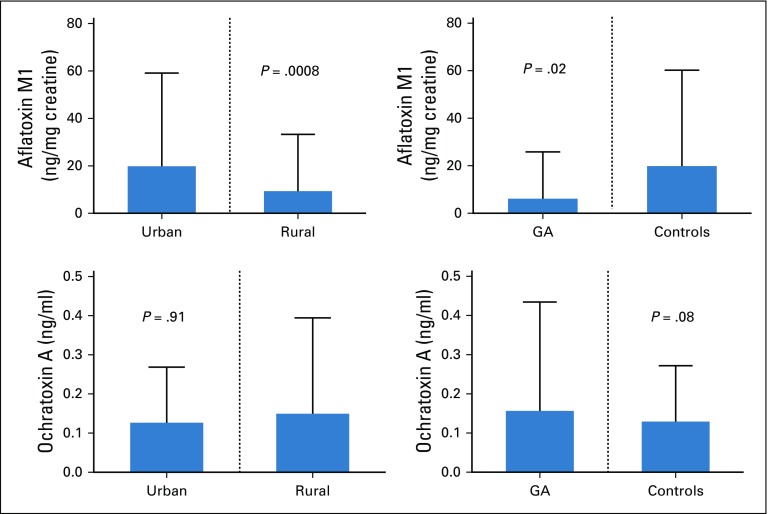
Urinary aflatoxin M1 and serum ochratoxin A stratified by residence and the presence of gastric adenocarcinoma (GA).

Of 254 patients with aflatoxin M1 results, 149 (63%) had detectable toxin in their urine. The median urinary aflatoxin M1 level was 33 ng/mL (IQR, 22-53 ng/mL); after correcting for urine creatinine, the median level was 35 ng/mg creatinine (IQR, 11- 397 ng/mg creatinine). This was not dependent on age (*P* = .38) or sex (*P* = .37) but was significantly higher in patients living in urban areas (Fig 4). Having aflatoxin in urine was not affected by lack of basic household goods (*P* = .40) or good housing (*P* = .34). Of the patients with ochratoxin results, 278 (96%) of 289 had evidence of ochratoxin A in their blood. The median level was 0.1 ng/mL (IQR, 0.06-0.16 ng/mL). There was no significant difference in ochratoxin levels for patients living in rural or urban areas (Fig 4). Similarly, ochratoxin levels were not different between patient cases and controls (Fig 4). Age, sex, and socioeconomic class had no influence on ochratoxin levels (*P* = .65, .56, and .82, respectively).

## DISCUSSION

In this study, gastric cancer was associated with low socioeconomic status, frequent exposure to biomass smoke (an effect restricted to intestinal-type cancers), and regular consumption of processed meat. Regular consumption of fruits and vegetables was protective against gastric cancer, and exposure to biomass smoke was associated with evidence of oxidative DNA damage in controls.

As previously published, the median age for patients with gastric cancer was at least a decade lower than that reported from developed countries, with 20% of the patients being under the age of 45 years (early-onset cancers).^6^ The prevalence was not significantly different between males and females, which is another point of distinction from industrialized countries, where male patients predominate.^1^ Biomass smoke was associated with gastric cancer, a finding that requires further corroboration. Controls exposed to biomass smoke had significantly higher levels of urinary 8-OHdG and 8-isoprostanes than those not frequently exposed, suggesting a link between biomass smoke and oxidative stress. This is consistent with a Norwegian study that showed that oxidative damage to DNA and repair was induced by wood smoke particles in human A549 and THP-1 cell lines.^23^ Cigarette smoking is a well-described risk factor for gastric cancer.^24,25^ Some of the carcinogenic compounds that are found in cigarette smoke (eg, benz[a]anthracene and benzo[a]pyrene) are also found in biomass smoke.^26^ There may be molecular parallels between swallowed biomass smoke and inhaled tobacco smoke, both of which increase cancer risk by inducing oxidative stress in epithelial cells.

Measured urinary levels of 1-OHP, a metabolite of polycyclic aromatic hydrocarbons (PAHs), were not associated with gastric cancer but were increased with exposure to biomass smoke in a stepwise manner. 1-OHP is not the best biomarker for assessment of long-term biomass smoke exposure because metabolism of PAH is rapid, and therefore, checking for metabolites in urine might not necessarily be an accurate reflection of such exposure in patients whose cancer has already developed and who would already have adapted to severe ill health. Changes in behavior when gastric cancer developed could have led to reduced exposure to biomass smoke, resulting in lower 1-OHP levels, perhaps because ill health reduces activities of daily living. In addition, measured PAH metabolites could have also come from other sources such as fumes from diesel engines, resulting in higher readings in patients not reliant on biomass fuels. This might be especially true in urban areas or near main roads. A study cohort including 256,357 men in Sweden showed that there was an increased risk of gastric cancer in workers exposed to diesel fumes.^27^ In addition, a 15-year United Kingdom (UK) cohort study involving more than 34 000 employees in eight UK oil refineries found that gastric cancer risk was increased in laborers with long service compared with the UK general population.^28^ All these data illustrate the need for further research on environmental pollution and gastric cancer.

Studies from developed countries have linked gastric cancer to low socioeconomic status.^29^ In this study, patients with gastric cancer had less basic household items and were living in poorer-quality houses without ready access to piped or treated water. This effect remained significant after adjusting for rural residence, which is where many of the patients lived. We believe that the effect of poverty may operate through some of the risk factors we have identified. Many Zambians do not eat fruit on a daily basis, and the widely available or affordable fruits are predominantly seasonal. These data add more evidence in support of the protective properties of regular fruit intake. Some investigators have reported a benefit of *Allium* vegetables, such as onions, for protection against gastric cancer.^30,31^ Our data did not show any significant protection of *Allium* vegetables against gastric cancer probably because of the low number of patients not eating onions on a daily basis. Regular consumption of eggplants (aubergines) was found to reduce the odds of having gastric cancer. Eggplants are not part of the traditional Zambian diet, but their consumption is increasing, particularly in urban areas. They are solanaceous plants containing glycoalkaloids, which are believed to have some anticarcinogenic properties.^32^ This could explain their negative association with gastric cancer.

The proportion of patients with detectable aflatoxin M1 in their urine was high, particularly for those living in urban areas. It could be an indication of poor grain storage, especially maize, which is the staple food in Zambia. Socioeconomic status did not show any influence on exposure to aflatoxins, suggesting that the source of the toxin could also be from well-packaged commercially available maize and groundnut products consumed by urban dwellers, rich or poor alike. Patients with gastric cancer had significantly lower aflatoxin M1 levels than the controls. This could be a result of changes in food intake as a result of the illness itself. In addition, the metabolism of aflatoxins is quite rapid, and the assay we used was only validated to determine exposure of aflatoxin ingestion in the prior 2 to 3 days.^33^ Exposure to ochratoxins was high in this patient group. The proportions found were much higher than those reported among Koreans (42%).^34^ However, unlike the aflatoxins, there was no significant difference between urban and rural residents or between patient cases and controls.

Despite the known limitations of a hospital-based study, mapping the town of permanent residence showed a fairly good representation of patients across the country, correlating well with the population distribution in Zambia. We do acknowledge that these data represent a convenience sample based on referral for EGD and not a population-based sample; therefore, they may not reflect epidemiology across the whole nation.

In conclusion, biomass smoke exposure is a risk factor for gastric cancer, possibly mediated through oxidative stress. Regular consumption of green vegetables and eggplants reduces the odds of developing gastric cancer, whereas regular consumption of processed meat increases the odds of developing gastric cancer.

## References

[1] Bray F Ferlay J Soerjomataram I et al Global cancer statistics 2018: GLOBOCAN estimates of incidence and mortality worldwide for 36 cancers in 185 countries CA Cancer J Clin 68 394 424 2018 30207593 10.3322/caac.21492

[2] Asombang AW Kelly P Gastric cancer in Africa: What do we know about incidence and risk factors? Trans R Soc Trop Med Hyg 106 69 74 2012 22136952 10.1016/j.trstmh.2011.11.002

[3] McFarlane G Forman D Sitas F et al A minimum estimate for the incidence of gastric cancer in Eastern Kenya Br J Cancer 85 1322 1325 2001 11720468 10.1054/bjoc.2001.1994 PMC2375245

[4] Laryea DO Awuah B Amoako YA et al Cancer incidence in Ghana, 2012: Evidence from a population-based cancer registry BMC Cancer 14 362 2014 24884730 10.1186/1471-2407-14-362 PMC4046022

[5] Kayamba V, Sinkala E, Mwanamakondo S, et al: Trends in upper gastrointestinal diagnosis over four decades in Lusaka, Zambia: A retrospective analysis of endoscopic findings. BMC Gastroenterol 15:127, 2015 10.1186/s12876-015-0353-8 PMC4596361 26444265

[6] Kayamba V Asombang AW Mudenda V et al Gastric adenocarcinoma in Zambia: A case-control study of HIV, lifestyle risk factors, and biomarkers of pathogenesis S Afr Med J 103 255 259 2013 23547703 10.7196/SAMJ.6159 PMC3815678

[7] Asombang AW, Kayamba V, Turner-Moss E, et al: Gastric malignancy survival in Zambia, Southern Africa: A two year follow up study. Med J Zambia 41:13, 2014 PMC10511215 37731812

[8] Fernando N Holton J Zulu I et al *Helicobacter pylori* infection in an urban African population J Clin Microbiol 39 1323 1327 2001 11283050 10.1128/JCM.39.4.1323-1327.2001 PMC87933

[9] World Cancer Research Fund: Continuous Update Project: Stomach cancer. https://www.wcrf.org/dietandcancer/stomach-cancer

[10] Abnet CC Corley DA Freedman ND et al Diet and upper gastrointestinal malignancies Gastroenterology 148 1234 1243.e4 2015 25680671 10.1053/j.gastro.2015.02.007 PMC4414068

[11] Reference deleted

[12] Yard EE Daniel JH Lewis LS et al Human aflatoxin exposure in Kenya, 2007: A cross-sectional study Food Addit Contam Part A Chem Anal Control Expo Risk Assess 30 1322 1331 2013 23767939 10.1080/19440049.2013.789558 PMC3725670

[13] Shirima CP Kimanya ME Kinabo JL et al Dietary exposure to aflatoxin and fumonisin among Tanzanian children as determined using biomarkers of exposure Mol Nutr Food Res 57 1874 1881 2013 23776058 10.1002/mnfr.201300116 PMC4044922

[14] Ezekiel CN Warth B Ogara IM et al Mycotoxin exposure in rural residents in northern Nigeria: A pilot study using multi-urinary biomarkers Environ Int 66 138 145 2014 24583186 10.1016/j.envint.2014.02.003

[15] Bonjour S, Adair-Rohani H, Wolf J, et al: Solid fuel use for household cooking: Country and regional estimates for 1980-2010. Environ Health Perspect 121:784-790, 2013 10.1289/ehp.1205987 PMC3701999 23674502

[16] Kayamba V Bateman AC Asombang AW et al HIV infection and domestic smoke exposure, but not human papillomavirus, are risk factors for esophageal squamous cell carcinoma in Zambia: A case-control study Cancer Med 4 588 595 2015 25641622 10.1002/cam4.434 PMC4402073

[17] Mota OM Curado MP Oliveira JC et al Risk factors for esophageal cancer in a low-incidence area of Brazil Sao Paulo Med J 131 27 34 2013 23538592 10.1590/S1516-31802013000100005 PMC10852069

[18] Mlombe YB Rosenberg NE Wolf LL et al Environmental risk factors for oesophageal cancer in Malawi: A case-control study Malawi Med J 27 88 92 2015 26715952 10.4314/mmj.v27i3.3 PMC4688868

[19] Kullberg C Andersson T Gustavsson P et al Cancer incidence in Stockholm firefighters 1958-2012: An updated cohort study Int Arch Occup Environ Health 91 285 291 2018 29164319 10.1007/s00420-017-1276-1 PMC5845066

[20] Reference deleted

[21] Reference deleted

[22] Reference deleted

[23] Danielsen PH Loft S Kocbach A et al Oxidative damage to DNA and repair induced by Norwegian wood smoke particles in human A549 and THP-1 cell lines Mutat Res 674 116 122 2009 19041418 10.1016/j.mrgentox.2008.10.014

[24] Xu Z Qi F Wang Y et al Cancer mortality attributable to cigarette smoking in 2005, 2010 and 2015 in Qingdao, China PLoS One 13 e0204221 2018 30235293 10.1371/journal.pone.0204221 PMC6157816

[25] Praud D, Rota M, Pelucchi C, et al: Cigarette smoking and gastric cancer in the Stomach Cancer Pooling (StoP) Project. Eur J Cancer Prev 27:124-133, 2018 10.1097/CEJ.0000000000000290 27560662

[26] Kayamba V Heimburger DC Morgan DR et al Exposure to biomass smoke as a risk factor for oesophageal and gastric cancer in low-income populations: A systematic review Malawi Med J 29 212 217 2017 28955435 10.4314/mmj.v29i2.25 PMC5610298

[27] Sjödahl K Jansson C Bergdahl IA et al Airborne exposures and risk of gastric cancer: A prospective cohort study Int J Cancer 120 2013 2018 2007 17266028 10.1002/ijc.22566

[28] Rushton L Alderson MR An epidemiological survey of eight oil refineries in Britain Br J Ind Med 38 225 234 1981 7272234 10.1136/oem.38.3.225 PMC1008879

[29] Barker DJ Coggon D Osmond C et al Poor housing in childhood and high rates of stomach cancer in England and Wales Br J Cancer 61 575 578 1990 2331444 10.1038/bjc.1990.129 PMC1971379

[30] You WC Blot WJ Chang YS et al *Allium* vegetables and reduced risk of stomach cancer J Natl Cancer Inst 81 162 164 1989 2909758 10.1093/jnci/81.2.162

[31] Zhou Y Zhuang W Hu W et al Consumption of large amounts of *Allium* vegetables reduces risk for gastric cancer in a meta-analysis Gastroenterology 141 80 89 2011 21473867 10.1053/j.gastro.2011.03.057

[32] Friedman M Chemistry and anticarcinogenic mechanisms of glycoalkaloids produced by eggplants, potatoes, and tomatoes J Agric Food Chem 63 3323 3337 2015 25821990 10.1021/acs.jafc.5b00818

[33] Smith LE Mbuya MNN Prendergast AJ et al Determinants of recent aflatoxin exposure among pregnant women in rural Zimbabwe Mol Nutr Food Res 61 1601049 2017 10.1002/mnfr.201601049 28544789

[34] Jung S Choe B Choi E et al Survey of mycotoxins in commonly consumed Korean grain products using an LC-MS/MS multimycotoxin method in combination with immunoaffinity clean-up Food Sci Biotechnol 24 1193 1199 2015

